# Cross-Sectional Analysis of Taiwanese Pharmacy Students’ Experiences and Perceptions of Transitioning from In-Hospital Internships to Distance Learning Due to COVID-19

**DOI:** 10.3390/healthcare10081369

**Published:** 2022-07-23

**Authors:** Shu-Fen Huang, Chin-Wei Hsu, Chia-Li Lin, Yen-Ling Ko, Hui-Chen Su

**Affiliations:** 1 Department of Pharmacy, Chi Mei Medical Center, Tainan 710, Taiwan; pharmacyh0520@gmail.com (S.-F.H.); u103534001@gmail.com (C.-W.H.); 2 Graduate Institute of Adult Education, National Kaohsiung Normal University, Kaohsiung 802, Taiwan; linchiali0704@yahoo.com.tw; 3 Department of Internal Medicine, Chi Mei Medical Center, Tainan 710, Taiwan; dreamyfairy79@hotmail.com; 4 Department of Pharmacy, Chia Nan University of Pharmacy & Science, Tainan 717, Taiwan

**Keywords:** Coronavirus Disease 2019, hospital pharmacist, introductory pharmacy practice experiences (IPPE), pharmacy education, distance learning

## Abstract

The introductory pharmacy practice experiences (IPPE) in Taiwan, which are traditionally conducted in physical hospital settings, incorporated up to 30% distance learning from May 2021 due to Coronavirus Disease 2019 (COVID-19). A web-based cross-sectional survey was adopted to investigate pharmacy students’ experiences and perceptions of transitioning from in-hospital internships to distance learning due to COVID-19 in the pharmacy department of a university in Southern Taiwan. We analyzed the results to discover factors that significantly affected students’ perceptions of transitioning from in-hospital internships to distance learning. In total, 81 interns from the university’s pharmacy department responded to the questionnaire. Approximately half of the participants felt happy when they learned, before the internship began, that the internship would be partially replaced with distance learning. The overall satisfaction rate was 67.9%, and no significant differences was observed in students’ satisfaction between hospital size or distance-learning time. However, more students in the medical center felt they had insufficient time to finish assignments compared to those in the regional hospitals, and the students who had 11–15 days of distance learning felt that they interacted more smoothly with their peers compared to those who had other durations. Program designers should make distance internship courses more student-centered, with a focus on increasing interactions between students, teachers, and peers to compensate for the lack of physical presence.

## 1. Introduction

The World Health Organization (WHO) declared Coronavirus Disease 2019 (COVID-19) a global pandemic on 11 March 2020 [1,2]. To control the spread of COVID-19, many countries began adopting non-pharmaceutical interventions, such as social distancing, isolation, lockdowns, and school closures; all of which had a significant effect on people’s daily lives [3,4]. Moreover, according to the United Nations Educational, Scientific, and Cultural Organization (UNESCO), approximately half of the world’s student population was affected by school closures in 2020 [5].

Taiwan controlled the spread of COVID-19 with strict travel restrictions and quarantines in the early stages, which minimized the impact of the global outbreak [6]. However, on 19 May 2021, due to the sharp rise in domestic infection cases, the Central Epidemic Command Center (CECC) announced that Taiwan had entered the third level of alert response. All meetings were prohibited, and gatherings were restricted to fewer than five people indoors and fewer than ten outdoors [7,8]. Consequently, schools were forced to close and students across the country switched to online-learning platforms. Moreover, various internship programs were canceled or postponed to avoid viral transmission amongst students.

COVID-19 also had a significant impact on higher education in the field of pharmacy, with the closure of pharmacy schools, and students and teachers switching to online teaching platforms. Pharmacy internships, including introductory pharmacy practice experiences (IPPE) and advanced pharmacy practice experiences (APPE), were also affected. The hospitals that provided this training were often located in the areas that were most severely affected by the pandemic and were unable to continue onsite training for students because of COVID-19 regulations. However, to allow pharmacy students to complete their internships, some units allowed their students to transition to online/virtual internships, while others provided a hybrid of physical and virtual internships, which enabled their students to practice remotely as well as engage in face-to-face learning and discussions with teachers at the hospital [9,10].

Pharmacy students in Taiwan are required to complete a 640-hour IPPE in a pharmacy department at a qualified teaching hospital to obtain a practical understanding of drug dispensing, drug-management concepts, drug consultation, clinical pharmacy services, etc., to register for the Professional and Technical Examination of Pharmacists held by the Ministry of Examination. Appropriate hospitals arrange the content and scheduling details of the internship in accordance with the regulations of the national examination authority, and the school arranges for teachers that visit students during the internship [8,11]. The pharmacy department of the school summarizes the number of pharmacy interns that qualified teaching hospitals can train and then distributes students according to their aspirations or school achievements. In response to the third wave of COVID-19 in China, and to reduce the risk of cluster infection, a suspension order was deployed that altered the original requirement that pharmacy students must complete 100% of their physical internships in hospitals. Furthermore, to protect the right of students to participate in the licensure examination as scheduled, the Ministry of Education, after convening a meeting of various pharmacy schools in Taiwan, decided that practice hospitals could arrange up to 30% of the 640-hour IPPE (192 h) as distance learning in place of the physical internship; the mode of distance learning could be online, and either synchronous or asynchronous. It could also be conducted via a virtual classroom, depending on the availability of the practice hospital’s hardware and software equipment and the arrangements made to meet the teaching objectives. However, Taiwanese hospitals had little experience of adopting distance learning to replace physical internships. Moreover, to the researchers’ best knowledge, no research has examined how to arrange the contents of remote practice in Taiwanese hospitals.

Many studies have confirmed that students’ perceptions and opinions are important references for the arrangement of educational programs [12,13,14], and collaboration between students and clinical faculties at internship hospitals can create effective educational programs [15]. Instructional media designed from the perspective of participants can help them follow instructions more effectively [16]. Owing to the COVID-19 pandemic prevention policy, many hospitals needed to offer a hybrid live/virtual pharmacy internship. This presented a challenge for faculties and course designers who were forced to think creatively in the delivery of learning activities [6]. The association between students’ perceptions of distance learning at IPPE and their acceptance of distance learning before their internship should be surveyed [17]. Yu et al. noted that a lack of preparation for the sudden shift to the delivery of online team-based learning led to decreased student motivation and engagement [18]. This study focuses on collecting and exploring the perceptions and suggestions of Taiwanese pharmacy students regarding distance learning as a partial replacement for physical internships.

## 2. Materials and Methods

### 2.1. Study Design

The web-based survey is an effective way to understand the thoughts of a large number of students in a limited time [12,19]. We conducted a web-based cross-sectional survey investigating pharmacy students’ experiences and perceptions of transitioning from in-hospital internships to distance learning due to COVID-19 in the pharmacy department of a university in Southern Taiwan.

### 2.2. Survey Development

The questionnaire was developed by a senior faculty member with at least 10 years of experience in teaching pharmacy interns, and was approved by the Academic Affairs Committee of the Department of Pharmacy. A brief description of the purpose of the survey and the anonymity and voluntary nature of the questionnaire was included at the beginning of the questionnaire. It was divided into two sections: (1) students’ backgrounds and acceptance of distance learning before the internship, and (2) students’ perceptions and suggestions regarding the transition from in-hospital internships to distance learning. Twenty-one close-ended and four open-ended questions were included. A five-point Likert scale was used to assess satisfaction levels.

### 2.3. Participants

Pharmacy students in Taiwan are usually assigned to different teaching hospitals for IPPE in the year before graduation. Participants were considered eligible for this study if they were enrolled in an IPPE internship in the pharmacy department of a university in Southern Taiwan from June to October 2021. The exclusion criterion was students not enrolled in a remote internship due to some hospitals postponing internships to maintain a full physical practice. The questionnaire included a preliminary question asking participants if there were distance-learning arrangements at the internship hospital. Participants who answered ‘No’ were shown a ‘thanks for the reply’ page and were not allowed to continue the questionnaire. The possible number of students was 114.

### 2.4. Data Collection

The perceptions and experiences of students were assessed using a web-based Google Forms questionnaire. The questionnaire was delivered through the office of the pharmacy department at the university during the last month of the internship.

### 2.5. Analysis

Multiple statistical analyses were performed using IBM SPSS version 27. Descriptive analysis was conducted to determine the participants’ characteristics. The continuous variable ‘age’ was reported as mean ± standard deviation (SD). Categorical outcomes (i.e., gender, self-assessment in the face of internship pressure, internship hospital grade, distance learning duration, highest percentage of distance-learning content, synchronized teaching ratios, acceptance of distance learning, and perceptions after the remote internship) were reported as numbers and percentages. The participants were required to rate their degree of agreement or satisfaction on a Likert scale ranging from 1 (strongly disagree) to 5 (strongly agree). Cronbach’s alpha coefficients were calculated to examine the internal consistency of the items of the questionnaire. The closer the Cronbach alpha was to 1.00, the greater the internal consistency of variables within the scale. The minimum acceptable level of Cronbach’s alpha was 0.7, as suggested by Nunnally [20]. Open-ended questions followed some of the questions to allow the researcher to understand what students thought about their responses. We were concerned about whether different hospital sizes and different distance learning durations would affect student satisfaction. Because the sample size of each group was expected to be small, non-parametric testing was used, namely the Mann–Whitney U test for comparing two groups, and the Kruskal–Wallis ANOVA test for a greater number of groups. The Mann–Whitney U test was used to determine the difference in satisfaction between the medical center and regional hospitals. The Kruskal–Wallis test was used to determine the difference in satisfaction between different distance-learning durations; for the statistically significant results of multiple pairwise comparisons, the significance level of the test was adjusted by Bonferroni correction to identify the groups in the sample that significantly differed. Probability values of *p* < 0.05, determined from two-sided tests, were considered statistically significant.

## 3. Results

Eighty-one questionnaires were collected, with a response rate of 71%. Cronbach’s alpha was used to test the reliability of the data collected from the questionnaires. The resulting reliability level was 0.816, higher than the suggested level of 0.7. The participants’ background information is shown in Table 1.

The average age of the students who completed the questionnaire was 23.5 ± 2.0 years, and women accounted for the majority of the sample (64.2%). Most of the students rated the pressure of their internship as ‘mild, but without assistance’ (58%), followed by ‘mostly as usual’ (33.3%) and ‘moderate and needed assistance’ (1.2%, only one participant). Most internships were held in the medical center (84%). The maximum number of remote-teaching hours arranged by the practice hospitals was 11–15 days (33.3%), and the highest proportion of distance-teaching content was clinical pharmacy services (58%), followed by drug consultation (22.2%). More than 60% of the distance learning was conducted via synchronous learning, which means that students learned the same topics at the same time online with the teacher. For example, in the communication course, students were asked to role-play with a patient, which would only have been possible with synchronous learning. Before engaging in this learning format, the students self-assessed their acceptance of distance learning, and more than 80% agreed that it was easy to find a space to avoid being disturbed, which was suitable for distance learning, and that they could ensure smooth internet speed for online distance learning. However, only approximately half of the students were happy to learn before the internship that part of their internship would be replaced by distance learning.

Concerning the student responses to the distance-learning experience questionnaire, only a small number of students (*n* = 11, 13.6%) believed that the distance-learning arrangement was much worse than they had imagined at the beginning. Regarding asynchronous teaching (*n* = 71, 87.7%) and the online assessment tool (*n* = 63, 77.8%), most of the students agreed that it was easy to use. Approximately 70% of the students agreed that interaction with teachers was as smooth as in physical lessons (*n* = 54, 66.7%), but fewer than half agreed that interaction with peers was as smooth as in physical courses (*n* = 39, 48.1%). Most of the students were satisfied with the time allotted (*n* = 53, 65.4%) and the information (*n* = 62, 76.5%) provided by their teachers to complete the distance-learning assignments. Overall, approximately 70% of the students (*n* = 55, 67.9%) were satisfied with the replacement of part of the required number of internship hours with distance learning (as shown in Table 2).

Furthermore, from the students’ suggestions to teachers and course planners, we found that only 19.8% agreed that the distance-learning teachers could continue lectures as they wished and did not often need to ask the students if they had any questions. Moreover, 72.8% of the students agreed that teachers should use multiple interactive methods (such as randomly asking students to answer questions, turning on their camera, and following the teacher’s instructions with hand gestures; answering questions in chat rooms; using the web-based white-board according to the teacher’s instructions, etc.) to facilitate learning. Additionally, 81.5% of the students felt that the internship units needed to delineate the distance-learning objectives (as shown in Table 3).

We wanted to determine or explore whether conditions such as ‘internship hospital grade’ and ‘distance-learning duration’ affected the interns’ perceptions of and overall satisfaction with distance learning. For the internship hospital grade, since there were only two interns in the community hospital, only the difference in the feelings and satisfaction of the intern students in the medical center and the regional hospitals were determined. The Mann–Whitney U test found no difference in overall satisfaction but found significant differences in ‘to complete the assignments/reports/quizzes specified in distance learning, the time given by the teacher is sufficient’. Table 4 also shows that there were differences in student satisfaction with the time given to complete the assignments in the medical center compared to the regional hospitals.

With regard to the distance-learning duration, different hospitals had different considerations when providing particular durations of distance learning, from less than two weeks to four weeks. We wanted to determine whether student satisfaction varied according to differences in course length. Consequently, we used the Kruskal–Wallis test to validate this notion and found no difference in overall satisfaction, although we did find a significant difference for ‘in synchronous distance teaching, the interaction with peers is as smooth as in the physical course’. A more in-depth analysis showed that the students in the 11–15-days group had better interactions with their peers than those in the 10-days group and the 16–20-days group (as shown in Table 5).

## 4. Discussion

This study focused on collecting and exploring the experiences and suggestions of Taiwanese pharmacy students regarding distance learning as a partial replacement for physical internships. Most of the participants could handle the pressure of internship as usual or felt mild pressure, but without seeking assistance from others. The majority of the students’ internships were carried out in the medical center, and the highest percentage of distance-learning courses was in clinical pharmacy services. The students also acknowledged having felt supportive of the shift to distance learning before the internship began. However, half of the students were dissatisfied with the fact that part of their internship had been replaced with distance learning. Most of the students provided positive feedback on their distance-learning experiences, but in terms of peer interaction, half of the students believed that such interactions were not as smooth in distance learning as on the physical course. The students’ feedback on distance learning included suggestions on how to make use of various methods to increase their interactions with their peers and teachers. Most of the students were satisfied with the distance-learning teaching content and goals and with the hours allocated to the distance-learning courses.

Compared to many other countries, Taiwan was more successful at controlling the spread of COVID; thus, in-hospital internships for pharmacy students did not undergo a complete shift to distance learning. However, the conditions in Taiwan were conducive to students transitioning to distance learning, considering that we found that 80.2% of the sample could easily find a space suitable for distance learning and 84% felt that Taiwan had sufficiently stable Internet speed to accommodate online distance learning; this was in contrast to Almohammed et al., who reported only 58.6% and 50.6% agreement, respectively [21]. Some students stated that the distance learning required an increased amount of attention and active participation; otherwise, the students felt, since there was no supervision of the distance learning, especially with closed cameras, that they might not achieve the same level of learning. Interestingly, the present study surveyed students’ acceptance of distance learning before beginning their courses and demonstrated that only half of the students were happy when they learned that part of their internship had been replaced with distance learning.

Further analysis revealed the reasons for students’ dissatisfaction with distance learning, including comments such as ‘the effect of distance teaching was not as good as that of the physical hands-on internship’. ‘distance learning was no different from class lectures’. ’the hands-on internship provided more efficient learning’. and ‘there were a lot of unnecessary assignments that took a lot of time, and it was inconvenient to carry out discussion with peers’. Almohammed et al. stated that even with high-tech virtual-reality teaching tools, students still worried about practicing independently in the real world; since they were not prepared to communicate with patients in the real world, students were worried about their lack of practical experience [21]. Muflih et al. found that more than 60% of pharmacy students believed that the barriers to distance learning included time-consuming courses [22], and the students in our sample also believed that extra time was needed to complete their distance-learning courses. For instance, assignment discussion was more inconvenient among group members in distance learning than in regular learning, and, similar to the findings of Almendingen et al., up to 90% of the participants agreed that, in distance learning, there was a lack of academic communication with peers [23]. During the COVID-19 pandemic, people’s sense of anxiety may have been exacerbated by the fear of contagion and of loved ones falling ill, or feelings of emptiness during quarantine. Therefore, students’ mental health should also be considered. Social support networks and the establishment of online support groups could also help students stay connected and promote good mental health at home during the pandemic [24].

In this study, which surveyed students’ acceptance of distance learning before their internships began, half of the students had a positive view of distance learning, mostly for reasons such as ‘reduced commuting time and not needing to get up early’. ‘ability to balance the needs of pandemic prevention with learning efficiency, as the risk of infection in the hospital was reduced’. ‘online courses could be repeated’. ‘ability to concentrate on learning, not disturbed by classmates’. ‘learning at home was less stressful’. These reasons were similar to the findings of Altwaijry et al., in which students were satisfied because distance learning can be undertaken anywhere. Furthermore, the materials on online distance-learning courses can be repeatedly viewed, thereby increasing learning efficacy [25], Amir et al. also found that students were satisfied with having more time after class to review the teaching materials, which is an advantage of this learning format [26]. Therefore, if the threat of the pandemic decreases in the future, we can still consider using blended learning for internships.

Each internship hospital had its own arrangements for the duration of long-distance courses, ranging from less than two weeks to four weeks, but this did not affect the overall satisfaction of the students (as shown in Table 5). For 58% of the students, the subject of clinical pharmacy services was given the most time, followed by drug consultation. The reason these two subjects took so much time could be because they were not taught in schools, which means that the hospitals needed to devote more time to teaching this important information at the internship stage. The students also felt that the database search and the use of evidence-based medicine arranged during the distance-learning period were useful for the subsequent physical internship. However, some of the students stated that they had not thought about the need to use what they learned for several weeks afterwards. Since the distance-learning programs were concentrated on the first two weeks of the internship, the students had almost forgotten what they had learned by the second half. One student stated: ‘The hospital introduced the operating system and process during distance teaching, but it is difficult for a student who had not even entered the hospital to understand’. Another student claimed: ‘In one of the video courses, the teacher introduced the use of the medicine package machine to the students with a camera, but after the course, the students still would not use it’. Consequently, course planners should consider the course content and sequence of distance learning to avoid ineffective teaching.

Only 48.1% of the students agreed that ‘distance learning is the same as physical internship regarding peer communication’, which leads to the assumption that distance learning still features obstacles. For example, discussion courses could be held face-to-face in physical classrooms and libraries. Moreover, some students found it uncomfortable to use online platforms to discuss with their peers without being able to see or hear each other, since most of the students turned off their microphones or cameras and turned them on only when they want to speak. The inability to see each other’s faces or voices impaired peer interaction [27]. In addition to using online platforms, students also used messaging software for discussions (e.g., Line, WhatsApp), but these discussions were not always timely, especially if the other party was not online—the students had to wait until their peers saw the message and responded to continue the discussion [28]. Group-discussion course formats, such as team-based learning, might reduce students’ motivation and engagement levels if educators are not trained or prepared to switch from physical to online teaching [18]. Although this method can be used as an alternative in case conducting physical classes is not possible, students were less willing to discuss openly and provide timely feedback in distance learning, which might have affected their learning satisfaction and achievements.

Furthermore, only 66.7% of the students in this study agreed that interactions with their teachers during distance learning were as convenient as physical class learning, indicating that some of the students still believed that distance teaching was not as efficient as face-to-face teaching. Khalil et al. pointed out the following reasons for dissatisfaction with online courses: teachers’ unfamiliarity with troubleshooting poor Internet connections, microphone-volume issues, and other technical problems, which, they believed, negatively affected students’ learning experience [29]. Moreover, some students thought that non-verbal communication with teachers was very important for the learning process. Therefore, distance-learning teachers needed multiple interaction methods, such as interspersed question-and-answer sessions, mandatory camera-on discussions, chatroom discussions, and the use of whiteboards to enhance interaction with students. In this study, more than 70% of students agreed that multiple interactions could help with learning. Therefore, the success of distance learning also depended on teachers’ familiarity with hardware and software equipment and multiple interaction methods, as well as knowledge of using video-teaching tools.

Regarding the number of medical services available, the size of the medical center was larger than those of the regional hospitals, and the teaching resources were relatively abundant. However, the medical-center interns’ satisfaction with the statement, ‘to complete the assignments/reports/quizzes specified in distance teaching, the time given by the teacher is sufficient’, was significantly lower than that of the regional-hospital interns. It was speculated that the medical center arranged more remote assignments, resulting in greater time pressure on students to complete their assignments. This reminded us that distance-learning courses do not entail the direct transformation of physical courses into pre-recorded lectures; rather, the emphasis should be on promoting more direct interaction and feedback from students during online sessions.

As mentioned above, although the distance-learning duration allotted by each hospital varied greatly, there was no difference in overall satisfaction based on this aspect. Only ‘when teaching online, interaction with peers is as convenient as in a physical course’ varied significantly between groups. The reasons that students believed made interactions not as convenient as in the physical classroom were: ‘there were unacquainted interns from other schools, making conversation less desirable’. ‘online courses did not promote peer interactions’. ‘interference occurred when everyone turned on the microphone, and there may have been a lag in communication’. ‘some video software did not allow communication between students’, and ‘LINE groups might not provide timely feedback’. The students in the 11–15-days group felt that the interaction between peers was significantly smoother than that of the other two groups, probably because the teacher had a better opportunity to allow in-class student discussions. Further research should delve into the course content and how teachers could facilitate better peer-interaction opportunities.

This study had several limitations. (1) The study was conducted to investigate the perceptions of the pharmacy students of one university. More specific descriptions would have been available if the views of pharmacy students from other universities in Taiwan had been included. (2) The study only investigated the arrangement of the initial transition from in-hospital internships to distance learning in Taiwan from the students’ perspective; it did not examine the teachers’ planning of distance-learning content and their initial intentions. (3) The response rate was only 71%, probably because the students did not have the incentive to complete the questionnaire, or did not feel pressure associated with not completing it. Thus, the results of this study reveal some relevant information but cannot represent all students’ opinions. Future research should use both students’ and teachers’ perceptions to better understand how to arrange suitable distance learning to partially or fully replace in-hospital internships.

## 5. Conclusions

The COVID-19 pandemic has caused considerable changes in people’s working life and teaching models. The pharmacy internship in Taiwan, which was conducted in-person for years previously, has also changed. In 2021, Taiwanese hospitals were forced to transition from physical internships to online distance learning within a short preparation period. This study focuses on collecting and analyzing the experiences and suggestions of pharmacy students regarding the partial replacement of physical internships with distance learning. The majority (67.9%) of the participating students gave positive feedback on the distance internship programs arranged by pharmacy hospitals. Except for the time that the teachers allowed students to complete assignments and the smoothness of the students’ interactions with their peers, most of the satisfaction levels did not vary by internship hospital or by the number of days the remote internship lasted. When interns practice remotely, hospital pharmacists should give them more time to complete their assignments.

## Figures and Tables

**Table 1:** Background information on the students who answered the questionnaire.

Variables	Number of Persons (*n*)	Percentage (%)
Age (years)	23.5 (mean)	±2.0 (SD)
Gender		
Male	29	35.8
Female	52	64.2
Self-assessment in the face of the pressure of internship		
No pressure	6	7.4
It is the same as usual	27	33.3
Mild, no assistance required	47	58.1
Moderate, assistance required	1	1.2
Internship hospital grade		
Medical center	68	84.0
Regional hospitals	11	13.5
Community hospitals	2	2.5
Distance learning time		
Within 10 days or less	15	18.5
11–15 days	27	33.3
16–20 days	20	24.7
21–28 days	19	23.5
Highest percentage of distance-learning content		
Clinical pharmacy services	47	58.0
Medication consultation	18	22.2
Pharmaceutical management	6	7.4
Drug dispensing	10	12.3
Synchronized teaching ratios		
0–20%	9	11.1
21–40%	12	14.8
41–60%	14	17.3
61–80%	15	18.5
81–100%	31	38.3
Acceptance of distance learning *		
I easily find a space that is suitable and has no disturbance for distance learning	65	80.2
It is easy to have a smooth internet speed for distance learning	68	84.0
I was pleased when I learned that part of the internship had been replaced with distance learning	41	50.6

* The figures in this section are the number and proportion of students who agreed/were satisfied with the self-assessment or more, respectively.

**Table 2:** Students’ perceptions regarding the distance-learning portion of the internship.

Variables	Number of Persons (*n*) *	Percentage (%) *
The distance learning arranged by the hospital was much worse than I had imagined at the beginning	11	13.6
The asynchronous teaching course path the hospital provided is easy to use	71	87.7
The online assessment tools provided by the hospital, such as the e-learning process, are easy to use	63	77.8
During synchronous distance learning, interaction with teachers is as smooth as in physical lessons	54	66.7
During synchronous distance learning, interactions with peers are as smooth as in physical lessons	39	48.1
To complete the assignments/reports/quizzes specified in distance learning, the time given by the teacher is sufficient	53	65.4
To complete the assignments/reports/quizzes assigned when teaching remotely, the information given by the teacher is sufficient	62	76.5
Overall satisfaction	55	67.9

* The figures in the table are the number and proportion of students who agreed/were satisfied with the self-assessment or more, respectively.

**Table 3:** Students’ suggestions regarding distance-learning components of internships.

Variables	Number of Persons (*n*) *	Percentage (%) *
Students’ suggestions to designated preceptors		
I think distance-learning teachers may continue to lecture as they wish and do not need to ask students often if they have any questions	16	19.8
I think distance-learning teachers should use multiple interactive methods to help in teaching (such as randomly asking students to answer questions, turning on the camera, and following the teacher’s instructions with hand gestures; answering questions in chat rooms; using the web-based whiteboard according to the teacher’s instructions, etc.).	59	72.8
Students’ suggestions to program preceptors		
The internship unit needs to let me understand the content and the objectives of distance learning target	66	81.5

* The figures in the table are the number and proportion of students who agreed/were satisfied with the self-assessment or more, respectively.

**Table 4:** Differences in the satisfaction levels of students between the medical center and the regional hospital.

Variants	Medical Center *	Regional Hospital *	*p*
The asynchronous teaching course path the hospital provided is easy to use	38.7	48.3	0.154
The online assessment tools the hospital provided, such as the e-learning process, are easy to use	39.1	45.7	0.341
In synchronous distance learning, the interaction with teachers is as smooth as on the physical course.	38.5	49.5	0.118
In synchronous distance learning, the interaction with peers is as smooth as on the physical course	38.7	48.0	0.200
To finish the assignments/reports/quizzes in distance learning, the time given by the teacher is sufficient	37.8	53.6	0.026
To finish the assignments/reports/quizzes assigned in distance learning, the information given by the teacher is sufficient	38.1	51.6	0.055
The hospital’s distance-learning arrangement was much worse than I had imagined at the beginning	41.8	29.1	0.070
Overall satisfaction	39.8	41.4	0.821

* In addition to the *p*-value, the values in the column are the average of the ranks.

**Table 5:** Results of the Kruskal–Wallis test on the differences in satisfaction with distance-learning duration.

Variants	(1) Within 10 Days or Less *	(2) 11–15 Days *	(3) 16–20 Days *	(4) 21–28 Days *	*p*	Rank
The asynchronous teaching course path the hospital provided is easy to use	36.1	43.3	36.2	44.2	0.479	
The online assessment tools the hospital provided, such as the e-learning process, are easy to use	35.4	45.1	38.6	39.8	0.543	
When teaching remotely in sync, interaction with teachers is as smooth as in physical lessons	37.4	46.0	41.3	34.1	0.303	
When teaching remotely in sync, interactions with peers are as smooth as in a physical lesson	30.8	52.7	32.9	38.5	0.005	(2) > (1), (2) > (3)
To complete the assignments/reports/quizzes in distance learning, the time given by the teacher is sufficient	39.4	46.9	39.6	33.2	0.222	
To complete the assignments/reports/quizzes assigned when teaching remotely, the information given by the teacher is sufficient	36.5	45.2	36.8	40.7	0.510	
The hospital’s distance-learning arrangement was much worse than I had imagined at the beginning	38.6	40.1	45.9	36.9	0.603	
Overall satisfaction	37.1	42.8	39.3	41.1	0.866	

* In addition to the *p*-value, the values in the column are the average of the ranks.

## Data Availability

The data presented in this study are available on reasonable request from the corresponding author.
